# Cardiovascular effects of Glucagon-like peptide 1 (GLP-1) receptor agonists

**DOI:** 10.1186/s12933-014-0142-7

**Published:** 2014-10-22

**Authors:** Francisco Kerr Saraiva, Andrei C Sposito

**Affiliations:** Faculdade de Medicina, Pontifica Universidade Catolica de Campinas, Campinas, Brazil; Laboratory of Atherosclerosis and Vascular Biology (AteroLab), Cardiology Division, State University of Campinas Medicine School (Unicamp), Campinas, Sao Paulo 13084-971 Brazil

## Abstract

Patients with type 2 diabetes have a several-fold increased risk of developing cardiovascular disease when compared with nondiabetic controls. Myocardial infarction and stroke are responsible for 75% of all death in patients with diabetes, who present a 2-4× increased incidence of death from coronary artery disease. Patients with diabetes are considered for cardiovascular disease secondary prevention because their risk level is similar to that reported in patients without diabetes who have already suffered a myocardial infarction. More recently, with a better risk factors control, mainly in intensive LDL cholesterol targets with statins, a significant decrease in acute cardiovascular events was observed in population with diabetes. Together with other major risk factors, type 2 diabetes must be considered as an important cause of cardiovascular disease.

Glucagon like peptide-1 receptor agonists represent a novel class of anti-hyperglycemic agents that have a cardiac-friendly profile, preserve neuronal cells and inhibit neuronal degeneration, an anti-inflammatory effect in liver protecting it against steatosis, increase insulin sensitivity, promote weight loss, and increase satiety or anorexia.

This review is intended to rationally compile the multifactorial cardiovascular effects of glucagon-like peptide-1 receptor agonists available for the treatment of patients with type 2 diabetes.

## Introduction

When compared with nondiabetic controls, T2D patients have a several-fold increased risk of developing cardiovascular disease (CVD) [1]. At least, 68% of people >65 years of age with diabetes die of some form of CVD. Among adults with diabetes, CVD death rates are 2-4× higher than the rates for adults without diabetes [2]. Patients with T2D are considered for CVD secondary prevention because their risk level is similar to that reported in nondiabetic patients who have already suffered a MI [3]. More recently, with a better risk factors control, mainly in intensive LDL cholesterol targets with statins, a significant decrease in acute cardiovascular events was observed in diabetic population. Together with other major risk factors, T2D must be considered as an important cause of CVD. Indeed, from a cardiological point of view, “diabetes is a cardiovascular disease” [4]. Unfortunately, despite significant advances in anti-glycemic therapies, macrovascular complications are still the most common cause of death in T2D patients [5].

The observation that intrajejunal glucose promotes greater insulin release than intravenous glucose administration was reported 50 years ago by McIntyre et al. [6] Perley and Kipnis estimated the intestinal component accounts for 50%–70% of the total insulin secreted after an oral glucose load. The ‘incretin effect’ was coined by Creuzfeld and Ebert in 1985 to designate this phenomenon [7]. Nauck et al. [8] demonstrated that the incretin effect is impaired in patients with T2D. Recently, An et al. observed that the improvement in the glycemic control potentiates insulin secretion induced by oral glucose ingestion but does not change the insulin secretion after iv glucose infusion indirectly demonstrating that the incretin effect is dependent of glucose/food absorption by the intestines [9].

**Figure 1:**
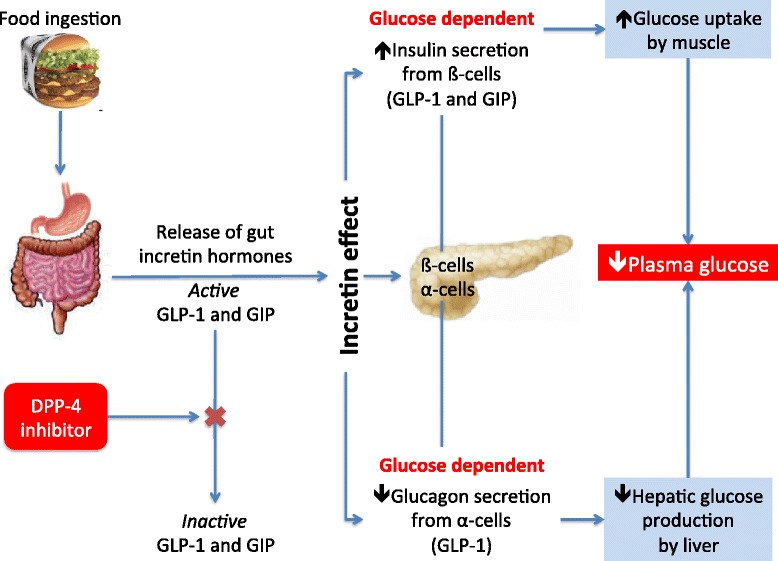
**GLP-1 is released from the small intestine after meal ingestion and enhance glucose-stimulated insulin secretion (** ***incretin action*** **).** DPP-4 rapidly converts GLP-1 and GIP to their inactive metabolites *in vivo*. Inhibition of DPP-4 activity prevents GLP-1 and GIP degradation, thereby enhancing incretin action (Adapted from Abrahamson MJ. The increting effect of GLP-1. Diabetes, Cardiovascular Disease and Stroke: Mechanisms and Risk Reduction. http://www.medscape.org/viewarticle/557239 and reference [14]).

Glucagon-like peptide 1 (GLP-1) and glucose-dependent insulinotropic polypeptide (GIP) are the two factors that account for most of the incretin effect [10]. GLP-1 and GIP are both secreted in response to food ingestion. GLP-1 is mainly produced in the enteroendocrine L cells located in the distal intestine exerting its effects through binding to the GLP-1 receptors (GLP-1R) expressed in the pancreas, heart, blood vessels, gastrointestinal tract (GIT), kidney, lung, breast, and central nervous system [11,12]. In the pancreatic ß-cell GLP-1, on elevated glucose concentrations, leads to stimulation of insulin secretion (Figure 1) [13].

Because the majority of the α-cells do not express GLP-1R, and also because GLP-1 inhibits glucagon secretion even in T1D patients with low insulin pancreatic reserve, the mechanism by which GLP-1 decreases glucagon secretion is still a matter of debate [15]. Recently, De Marinis et al. reported that GLP-1-induced suppression of glucagon release is dependent of protein kinase A (PKA) and independent of glucose or paracrine effects mediated by insulin or by somatostatin acting on somatostatin receptor subtype-2 [16,17].

Furthermore, GLP-1 decreases gastrointestinal motility extending the entry of nutrients to be absorbed by the GIT [18]. This effect seems to be very important for the normalization of postprandial glucose (PPG) elevations, and potentially even more important than the insulinotropic effects of GLP-1 for maintaining PPG homeostasis [19]. Many reports showed that GLP-1 effects involve neural modulation and peripheral effects, which increase satiety, resting energy expenditure and lower plasma concentrations of free fatty acids [20-22].

**Figure 2:**
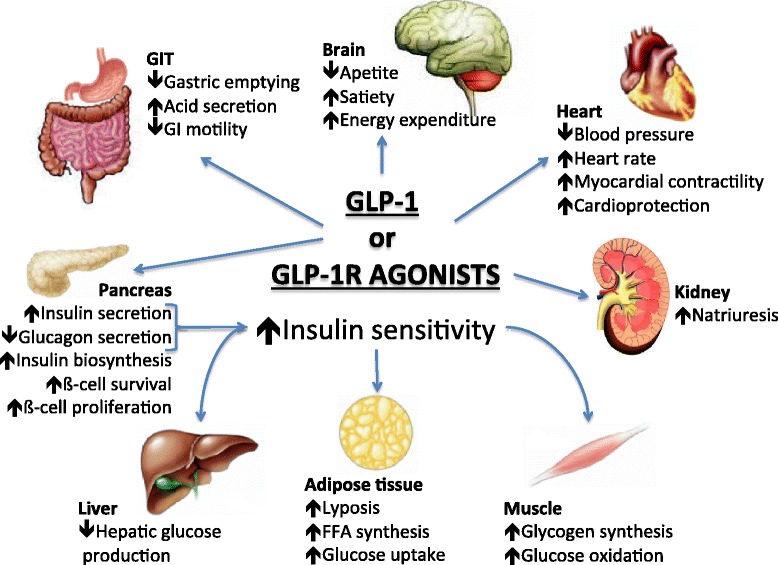
**Pleitropic effects of GLP-1 or GLP-1R agonists (Adapted from references** **[** 24 **]** **).**

Either GLP-1 or GIP potentiates glucose-dependent insulin response protecting β-cells against cytokine-induced apoptosis or glucose [23]. All of these GLP-1 effects (Figure 2) are potentially beneficial for patients with T2D, and have been sought exploited clinically with the development of GLP-1R agonists.

The phenomenon of induction of glucose sensitivity observed in glucose-resistant ß-cells is called glucose competence, i.e. the magnitude of the insulin secretion response to a rise in glucose concentration. More recently, Cornu et al. demonstrated in KO mice that the regulation of ß-cell number and function by GLP-1 depends on the cAMP/PKA mediated-induction of insulin-like growth factor-1 receptor (IGF-1R) expression and the increased activity of insulin-like growth factor 2 (IGF-2)/IGF-1R by an autocrine mode of action [25].

As depicted in Figure 1, dipeptidylpeptidase-4 (DPP-4) is an ubiquitously expressed transmembrane protein that removes N-terminal dipeptides from various substrate hormones, chemokines, neuropeptides and growth factors. DPP-4 inhibitors (DPP-4i) block degradation of GLP-1 and GIP, extend their insulinotropic effect, and improve glycemia [26].

Although the SAVOR and EXAMINE studies were delineated to verify cardiovascular safety of two DPP-4i, namely saxagliptin and alogliptin, respectively, both are considered to be the first controlled clinical trials to address the effect of GLP-1 system on CVD. In the SAVOR study DPP-4 inhibition with saxagliptin did not increase or decrease the rate of ischemic events, though the rate of hospitalization for heart failure was increased [27]. On the other hand, EXAMINE study showed that T2D patients, who had had a recent acute coronary syndrome, the rates of major adverse cardiovascular events were not increased with the DPP-4 inhibitor alogliptin as compared with placebo [28].

GLP-1R agonists represents a novel class of anti-hyperglycemic agent that improve health and survival of ß-cells (improvement in postprandial hyperglycemia), suppress glucagon (improvement in fasting hyperglycemia), improve insulin resistance (modest effect) and influence energy intake (augment satiety signal) with minimal, if at all, any side effects (weight neutral and non-hypoglycemic). The incretins address most of the proposed pathophysiologic mechanisms involved in the development of T2D (Figure 2). Besides the ß-cell dysfunction, incretin deficiency is now considered among the key factors in the pathophysiology of T2D. So, exogenous GLP-1R agonists have been more recently considered as a good choice for treating patients with diabetes [29].

In this brief review, we shall discuss recent important evidences of the potential clinical benefit of the incretin effect on cardiovascular system as well as the biological mechanisms, which underlie these effects.

### GLP-1R distribution in the cardiovascular system

GLP-1R is a member of the class B1 family of G protein-coupled receptor for which there are only predicted structures [30]. GLP-1R has been detected by reverse transcriptase-polymerase chain reaction in cardiac and vascular tissues isolated from both human and animal models [11,31], and their mRNA transcripts were demonstrated in the human heart [32].

GLP-1R protein has also been detected in human coronary artery endothelial cells (HCAEC) and human umbilical vein endothelial cells (HUVEC) [33,34]. Ban et al. detected GLP-1R protein expression by immunohistochemistry in mouse coronary endothelial and smooth muscle cells [35].

Many reports using polyclonal antisera were unable to detect GLP-1R protein in ventricular cardiomyocytes leaded to the revaluation of those earlier reports. Because of lack of sensitivity and problems with non-specificity of commercially available GLP-1R antisera detected multiple nonspecific immunoreactive proteins even in tissue extracts from Glp1r^−/−^ mice. Although Glp1r mRNA transcripts are detected in whole heart extracts such methods do not distinguish between vascular smooth muscle cells or atrial cardiomyocytes, there were technical difficulties in isolating pure cardiomyocyte populations from the ventricle without contamination of atrial cardiomyocytes isolated from neonatal mouse [36].

### Cardiac GLP-1R signaling pathways

**Figure 3:**
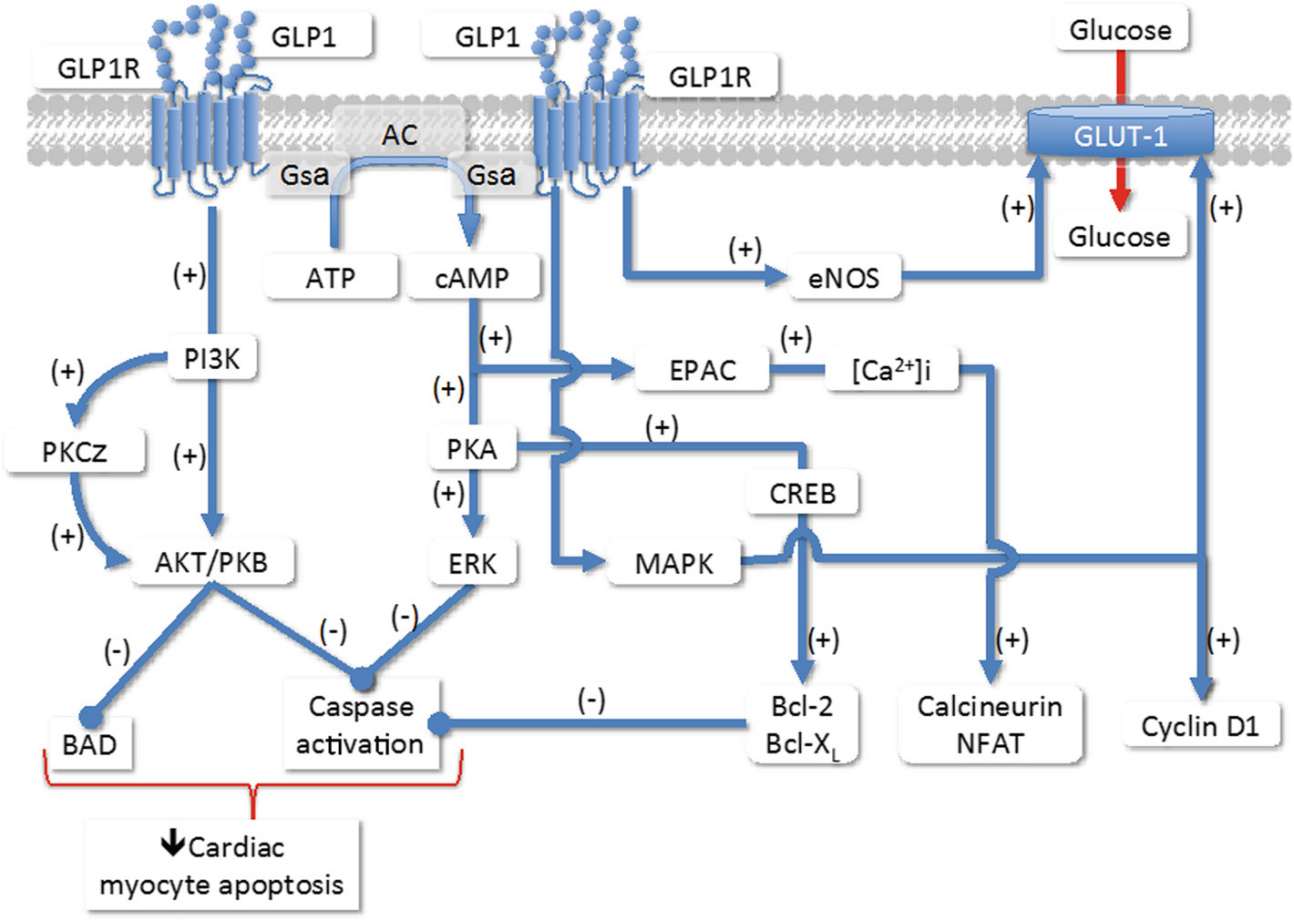
**Intracellular signaling pathways of GLP-1R in the cardiomyocytes (Adapted from references** **[** 36 **,** 39 **]** **).**

There is considerable overlap between pathways induced by the GLP-1R activation. As shown in Figure 3, GLP-1R couples to adenylyl cyclase leading to the activation of cAMP. Using a GLP-1 conjugated to tetramethyl rhodamine to monitor the internalization of the receptor, Kuna et al. [37] observed that after internalization, GLP-1R/ligand complex could be co-localized with adenylate cyclase in the endosome. Indeed, cAMP levels can be elicited by GLP-1 treatment of mouse cardiomyocytes without increasing intracellular Ca^2+^ or inducing contractility [38].

The involvement of cAMP/PKA/CREB pathway on decrease myocyte apoptosis has been unveiled by experiments prepared in cultured mouse cardiomyocytes. This function is mediated via the activation of the cAMP/PKA/CREB (cAMP-responsive element binding protein) and the transactivation of the EGF-R (epidermal growth factor receptor) leading to the activation of phosphatidylinositol-3 kinase (PI3K), protein kinase Cζ(PKCζ), Akt-protein kinase B (AKT/PKB), extracellular regulated kinase (ERK1/2 or mitogen-activated protein kinase [MAPK]) signaling pathways and to the up-regulation of the expression of the cell cycle regulator cyclinD1 (Figure 3). The increase in Akt and ERK phosphorylations lead to cardiomyocyte growth and activation of glucose metabolism [24,39-41].

The main mechanism of cardioprotective effects of GLP-1R agonists against oxidative stress-induced injury in H9c2 cells (cardiomyoblasts from Rattus norvegicus) and in cardiomyocytes is related to the scavenging of reactive oxygen species, by increasing the concentrations of endogenous antioxidant defenses and inhibiting cardiomyocyte apoptosis [42]. This cytoprotective effect is mediated by PI3K and partially dependent on ERK1/2 and seems to be glucose-independent. A protective role of retinoid X receptor is also related to hypoxia/reoxygenation injury in those cells [43]. Moreover, GLP-1 also inhibits palmitate- and ceramide-induced phosphatidylserine exposure and DNA fragmentation [44].

### Effects of GLP-1R activation in the heart

The observation that GLP-1 decreases contractility in primary culture of adult rat cardiomyocytes, despite increasing cAMP levels, [38] has also been reported in isolated rat hearts [45]. Studies in dogs demonstrated an increased myocardial glucose uptake during a hyperinsulinemic-euglycemic clamp [46].

Moreover, cardiometabolic effects of GLP-1 are attenuated in obesity and T2D, via mechanisms that may involve impaired p38-MAPK signaling. Using a swine experimental model, Moberly et al. confirmed and extended the observation, where GLP-1 significantly increased myocardial glucose uptake under basal conditions in lean humans, but this effect was impaired in T2D [47]. GLP-1 did not increase myocardial oxygen consumption or blood flow in humans or in swine, increasing p38-MAPK activity in lean, but not obese cardiac tissue [48].

Experimental studies in animals suggested that incretins may preserve cardiomyocyte viability, increase metabolic efficiency, inhibit the structural, and functional remodeling after MI [49]. Shannon’s group was the first to demonstrate that infusion of GLP-1(7-36) (1.5 pmol/kg/min) for 72 h in patients with left ventricular dysfunction (LVD) after MI improved global and regional left ventricular wall motion scores reducing hospital stay and in-hospital mortality [50,51].

GLP-1-mediated control of heart rate (HR) and blood pressure (BP) is complex and species specific. Synthetic human GLP-1 administered into the jugular vein of male rats acutely increased systolic and diastolic BP, as well as HR, which returned to basal levels 25 min after GLP-1 administration. The pre-treatment with propranolol or phentolamine did not prevent the increase in BP and HR. In rodents, these increases involve a dual pathways originating from both central nervous system and periphery with intact neural vagus transmission [52].

Intravenous infusion of GLP-1 for 48 h in healthy human subjects increased muscle sympathetic nerve activity but had no effect on BP, norepinephrine plasma concentration, or the sympathetic/parasympathetic balance as estimated by the HR variability, suggesting that the increase in sympathetic drive is at least partially compensated by an increase in the parasympathetic activity [53]. On the other hand, acute subcutaneous (SC) injection of GLP-1 increases HR and BP transiently in healthy human subjects, returning to normal ranges 50-60 min after injection [54]. A mean increase in HR of 1.86 beats/min was reported in a meta-analysis including available data from randomized controlled trials testing GLP-1 agonists against placebo in patients with T2D [53].

In the cardiovascular system, incretins have been recently associated to the increase of endogenous antioxidant defenses, inhibition of cardiomyocyte apoptosis, attenuation of endothelial inflammation and dysfunction [55].

### GLP-1R activation and artery wall function

GLP-1 has pleiotropic effects on the cardiovascular system. A GLP-1R agonist (liraglutide) increased endothelial nitric oxide synthase phosphorylation and nitric oxide (NO) production by the 5-AMP-activated protein kinase (AMPK)-dependent pathway and subsequent NO production in cultured HCAEC [56,57]. GLP-1 protects the cardiac microvessels against oxidative stress, apoptosis, and the resultant microvascular barrier dysfunction in diabetes, contributing to improvement of cardiac function and cardiac glucose metabolism. The protective effects of GLP-1 are dependent on downstream inhibition of Rho through a cAMP/PKA-mediated pathway [58].

As shown by Batchuluun at al., metformin and liraglutide improve high glucose-induced oxidative stress via inhibition of PKC-NADPH oxidase pathway in human aortic endothelial cells. All these effects were even more pronounced when both drugs were used together, suggesting a potential clinical benefit of this drug combination in decreasing the endothelial damage induced by hyperglycemia [59].

In humans, Kelly et al. showed that exenatide therapy for that 3-mo in patients with obesity and pre-diabetes had similar effects on microvascular endothelial function, markers of inflammation, oxidative stress, and vascular activation, as metformin. They assumed that improvements in endothelial function with GLP-1R agonists might be limited to the postprandial setting, particularly following the consumption of a high-fat meal [60].

GLP-1 enhanced acetylcholine-induced forearm blood flow but had no effect on blood flow induced by sodium nitro-prusside in healthy human subjects [61]. Patients with T2D with stable CAD presented an improvement in endothelial function expressed by an increase in flow-mediated vasodilation of the brachial artery, independent of changes in systolic and diastolic blood pressure during a hyperinsulinemic clamp in response to GLP-1 [35]. However, GLP- 1-mediated improvements in blood flow were considerably attenuated after a 2-month period of better glycemic control [62].

In obese patients, GLP-1 infusion significantly increased excretion of sodium (by 60%), calcium (by 60%) and chloride (by 44%) and significantly decreased excretion of H^+^ (by 75%). Besides these renoprotective qualities, these changes suggest that GLP-1 may have renoprotective qualities [63]. Moreover, Kim et al. demonstrated that cardiac GLP-1R expression is localized in cardiac atria and that GLP-1R activation promotes secretion of atrial natriuretic peptide (ANP) and increases BP [64].

In animal and cell models, GLP-1 has been shown to impact the development and/or progression of atherosclerotic plaques. Since GLP-1R has been localized by immunocytochemistry in mouse aortic smooth muscle cells, endothelial cells, monocytes, and macrophages, both direct and indirect actions of GLP-1 may contribute to the potential reduction of atherogenesis. Continuous infusion of exendin-4 in nondiabetic C57BL/6 and ApoE^−/−^ mice reduced monocyte adhesion to aortic endothelial cells and atherosclerotic lesion size after 40 days of treatment. The inflammatory markers monocyte chemoattractant protein-1 (MCP-1) and tumor necrosis factor α (TNF-α), were reduced by treatment with exendin-4 in response to lipopolysaccharide in cultured peritoneal macrophages harvested from mice [65]. However, circulating GLP-1 was found to be positively associated with total coronary load in humans in a fully adjusted model [OR: 2.53 (95% CI: 1.12-6.08; p = 0.03)], [66] but further studies are required to confirm these observations.

Continuous infusion of exendin-4 for 4 wk. in C57BL/6 mice reduced neointimal formation after endothelial denudation of the femoral artery [67]. Nagashima et al. [68] reported that continuous GLP-1 infusion reduced foam cell formation and the development of atherosclerotic lesions in ApoE^−/−^ mice. Recently, the same group demonstrated similar effects after infusion of liraglutide in apoE^−/−^ mice [69]. In addition to these findings on atherosclerotic plaque formation, GLP-1R agonists prevented the increase in plasminogen activator inhibitor type-1 (PAI-1) and vascular cell adhesion molecule-1 (VCAM-1) gene expression in response to TNF-α or hyperglycemia in HUVEC through PKA pathway [24,70].

### GLP-1R agonists effects on cardiovascular events

The first pieces of evidence of the potential clinical benefit of GLP-1 agonists have emerged recently, pointing to a decrease of major adverse cardiovascular and cerebrovascular events (MACCE) including stroke, MI, cardiac mortality, acute coronary syndrome, and revascularization procedures. A retrospective medical database analysis of T2D patients (n = 39,275) treated with exenatide or other glucose-lowering therapies (n = 381,218) indicated that the GLP-1 agonist might reduce in 19% the incidence of MACCE and in 12% cardiovascular hospitalizations [71]. Despite the absence of adverse cardiovascular effects has been confirmed, this benefit was not verified in an integrated analysis of 12 controlled, randomized, short-term clinical trials (12-52 weeks) comparing exenatide with placebo or insulin [72].

In both diabetic and non-diabetic patients presenting class II/IV heart failure, GLP-1 infusion led to an improvement of left ventricular (LV) ejection fraction, myocardial ventilation oxygen consumption, 6-min walk distance, and quality of life [50]. A study with infusion of exenatide in T2D patients with chronic heart failure reduced the pulmonary capillary wedge pressure and increased both inotropism and chronotropism [73]. These favorable results require further clinical trials in order to elucidate whether these effects will result or not in reduced mortality. In a study involving patients with CAD and preserved LV function, who were scheduled to undergo coronary artery bypass grafting (CABG), were randomized to receive standard therapy or treatment with GLP-1 (1.5 pmol/kg/min) as a continuous infusion beginning 12 h before CABG and continuing for 48 h after the procedure. The control group required greater use of inotropic and vasoactive infusions during the 48 h period after CABG to achieve the same hemodynamic results observed in the group receiving GLP-1. There were also more frequent arrhythmias requiring anti-arrhythmic agents in the control group [50].

In a non-randomized pilot study, Nikolaidis et al. investigated the safety and efficacy of a 72-h infusion of native GLP-1 combined to background therapy in 10 patients with acute MI and LV ejection fraction <40% (mean 29 ± 2%) after successful primary angioplasty, compared to 11 control patients. GLP-1 treatment was safe and elicited a significant improvement in LV ejection fraction (to a mean of 39 ± 2%) [74].

In a study involving 14 patients with normal ejection fraction and CAD awaiting revascularization, normal saline and native GLP-1 were infused on two different occasions from 30 min before until 30 min after a dobutamine stress echocardiography for evaluation of the global LV function; native GLP-1 significantly improved LV function at peak stress and at 30 min into recovery, predominantly in ischemic segments [75].

Fifty-eight patients with ST-segment-elevation MI and thrombolysis were randomized to receive either saline (n = 40) or exenatide (n = 18, 10 μg SC and 10 μg as an IV bolus 5 min before the onset of reperfusion plus 10 μg SC twice daily on the following 2 days) to evaluate whether this GLP-1R agonist could reduce the area of necrosis; exenatide administration significantly decreased release of CK-MB and troponin and reduced infarct size as evaluated by cardiac magnetic resonance after 1 month [14].

### Differences between GLP-1R agonists

GLP-1R agonists already in the market and others in development present the same mode of action and, having the same pleiotropic effects of native GLP-1, the differences in clinical profiles between them are related to differences in pharmacokinetics, structure and size of each formulation. The most remarkable clinically Important pharmacokinetic characteristic is the difference between the short-acting and continuous-acting compounds (Table 1) [13]. Indeed, continuous exenatide exposure once weekly elicited a greater response than did short-acting exenatide, improving glycemic and lipids controls and lipoprotein metabolism, and decreasing systemic inflammation. Indeed, as shown by Hermansen et al. liraglutide treatment in patients with T2D significantly reduced postprandial excursions of triglyceride and apo B48 after a fat-rich meal, independently of gastric emptying. Results indicate that liraglutide’s potential to reduce CVD risk via improvement of postprandial lipemia.

**Table 1:** **Effects of the short- and continuous-acting GLP-1R agonists (Adapted from reference** [13]**)**

	**Short-acting**	**Continuous-acting**
	Exenatide	Exenatide*
	Lixisenatide	Liraglutide
		Albiglutide
		Dulaglutide
		Semaglutide
Clinical effects		
GLP-1R activation	Intermittent	Continuous
HbA_1c_ reduction	**+**	**++**
FPG reduction	**+**	**++**
PPG reduction	**++**	**+**
Gastric emptying deceleration	**++**	**←→**
Body weight reduction	**++**	**++**
Blood pressure reduction	**+**	**+**
Hearth rate increase	**←→**	**+**

*Abbreviations*: *GLP-1R* glucagon-like peptide-1 receptor, *HbA*_*1c*_ glycated haemoglobin, *FPG* fasting plasma glucose, *PPG* postprandial glucose.

*Exenatide once-weekly.

Meloni et al. calculated the absolute benefit increase of using exenatide once week, GLP-1R agonist, vs an oral glucose-lowering medication or insulin glargina to achieve ADA-recommended goals. Exenatide once a week assisted more patients in reaching the majority of ADA-recommended therapeutic goals than treatment with sitagliptin, pioglitazone, or insulin glargine.

## Conclusions

Mechanistic and preliminary clinical evidence have consistently pointed toward beneficial effects of GLP-1 analogues and GLP-1R agonists on cardiovascular diseases in T2D patients. Indeed, a search at http://www.clinicaltrials.gov (as of, Sep ’14), employing cardiovascular disease and GLP-1 as keywords, returns 112 records comprising 42% completed, 39% recruiting, 8% not yet recruiting, 6% active, but not yet recruiting, 4% terminated, and 1% withdraw studies. Among those, 12% studies has results and 88% has no results available, which indicates towards an increasing interest in the cardiovascular effects of incretin-based therapies. The actual evaluation of cardiovascular benefits will be achieved with the completion of the Liraglutide Effect and Action in Diabetes: Evaluation of Cardiovascular Outcome Results (LEADER) trial, which enrolled 9,340 T2D patients randomized for treatment with liraglutide or placebo for an estimated period of 5 years (ClinicalTrials.gov Identifier: NCT01179048).

Tables 2 and 3 depict the most important results of GLP-1 and GLP-1R agonists from animal and human studies mentioned in the text.

**Table 2:** **Compilation of the most important results of GLP-1 and GLP-1R agonists from animal studies mentioned in the text**

	**GLP-1**	**References**	**GLP-1R agonist**	**References**
	Elicits cAMP in mouse cardiomyocytes	[38]	mRNA Expression is localized in cardiac atria and and its activation promotes secretion of atrial natriuretic peptide and increases BP.	[11,31,64]
	Inhibits palmitate- and ceramide-induced phosphatidylserine exposure and DNA fragmentation	[44]	Acts via cAMP in endosome	[37]
	Increases myocardial glucose uptake in dogs	[46]	Decreases myocyte apoptosis by activation of cAMP/PKA/CREB pathway	[39]
	May preserve cardiomyocyte viability, increases metabolic efficiency and inhibits the structural and functional remodeling after myocardial infarction.	[49]	Induces cardiomyocyte growth and activation of glucose metabolism by a mechanism envolving AKT and ERK phosphorylations	[24,40,41]
Animals studies	Increases systolic and diatolic BP, as well as HR im male rats acutely.	[52]	Has cardioprotective functions related to inhibition of cardiomyocytes apoptosis due their ROS scanvenger actions, by increasing endogenous antioxidant defenses.	[42]
	Inhibts glucagon release by a mechanism PKA dependent and glucose independent	[16,17]	Cardioprotective functions are mediated by PI3K and partially dependent on ERK1/2	[43]
	Decreases contractility in primary culture of adult rat cardiomyocytes and in isolated rat hearts	[45,76]	Attenuates atherosclerotic lesions by reducing monocyte/macrophage accumulation in the arterial wall and inhibits the inflammatory response in macrophages.	[65]
			Reduces the inflammatory markers: MCP-1 and TNF-α in response to lipopolysaccharide in cultured peritoneal macrophages harvested from mice.	[66]
			Reduces monocyte adhesion to aortic endothelial cells and atheroscleroticlesion size in nondiabetic C57BL/6 and ApoE−/− mice.	[65]

**Table 3:** **Compilation of the most important results of GLP-1 and GLP-1R agonists from human studies mentioned in the text**

	**GLP-1**	**References**	**GLP-1R agonist**	**References**
	Decreases gastrointestinal motility extending the entry of nutrients to be absorbed by the GIT	[18]	mRNA transcripts were demonstrated in the human heart.	[31]
	Normalizes postprandial glucose elevations by decreasing TGI motility, which seems to be more important than its insulinotropic effects.	[19]	Induces a mean increase in HR in patients with T2D.	[77]
	Improves endothelial function expressed by an increase in flow-mediated vasodilation of the brachial artery, independent of changes in systolic and diastolic blood pressure during a hyperinsulinemic clamp in patients with T2D with stable CAD.	[36]	Increases endothelial nitric oxide synthase phosphorylation and nitric oxide production by the AMPK-dependent pathway in cultured Human Coronary Artery Endothelial Cells.	[56,57]
	Increases myocardial glucose uptake under basal conditions in lean humans, but this effect was impaired in T2D.	[47]	With metformin ameliorates high glucose-induced oxidative stress via inhibition of PKC-NAD(P)H oxidase pathway in human aortic endothelial cells.	[59]
	improves global and regional LV wall motion scores reducing stay and in-hospital mortality of patients with LV dysfunction after myocardial infarction.	[50,51]	Increases endogenous antioxidant defenses, inhibits of cardiomyocyte apoptosis, attenuates of endothelial inflammation and dysfunction.	[62]
	Protects against cardiac microvascular injury in diabetes via a cAMP/PKA/Rho-dependent mechanism.	[58]	Reduces in 19% the incidence of major adverse cardiovascular and cerebrovascular events (MACCE) and in 12% cardiovascular hospitalizations.	[71]
	Enhances acetylcholine-induced forearm blood flow.	[61]	Reduces pulmonary capillary wedge pressure and increased both inotropism and chronotropism. In T2D patients with chronic heart failure.	[73]
Human studies	Increases muscle sympathetic nerve activity without affecting BP, norepinephrine plasma concentration, or the sympathetic/parasympathetic balance, where sympathetic drive is at least partially compensated by an increase in the parasympathetic activity	[62]	Reductes infarct size and improves subclinical LV function when added to primary percutaneous coronary intervention in patients with ST-segment-elevation myocardial infarction.	[14]
	May have renoprotective function by significantly increased excretion of sodium, calcium, and chloride and significantly decreased excretion of H + in obese patient.	[63]	When administered once in a week assistes more patients in reaching the majority of ADA-recommended therapeutic goals than treatment with sitagliptin, pioglitazone, or insulin glargine as shown by clinical trials.	
	Concentration in human plasma is found to be positively associated with total coronary plaque load.	[66]	When administered once in a week elicites a greater response than does short-acting exenatide once a day, improving glycemic and lipids controls, lipoprotein metabolism, and decreasing systemic inflammation.	
	Improves LVEF, myocardial ventilation oxygen consumption, 6-min walk distance, and quality of life. In both diabetic and non-diabetic patients presenting class II/IV heart failure	[78]		
	Achieves better glycemic control and comparable hemodynamic recovery without the requirements for high-dose insulin or inotropes when infused perioperatively in patients with CAD and preserved LV function scheduled to undergo coronary artery bypass grafting. There were also more frequent arrhythmias requiring anti-arrhythmic agents in the control group.	[79]		
	Treatment is safe and elicites a significant improvement LVEF in patients with acute MI and LVEF <40% after successful primary angioplasty when compared with control.	[74]		
	Protects the heart from ischemic LV dysfunction induced by dobutamine stress in patients with CAD.	[75]		

The potential cardiovascular benefits expected from this new therapeutic approach to obtain glycemic control in T2D arises a possibility to change the excessive cardiovascular burden related to this disease in both developed and developing countries worldwide.
